# A Theoretical and Clinical Framework for Parental Burnout: The Balance Between Risks and Resources (BR^2^)

**DOI:** 10.3389/fpsyg.2018.00886

**Published:** 2018-06-12

**Authors:** Moïra Mikolajczak, Isabelle Roskam

**Affiliations:** Research Institute for Psychological Sciences, Université Catholique de Louvain, Louvain-la-Neuve, Belgium

**Keywords:** parental burn-out, exhaustion, antecedent, cause, etiology, theory, model, measure

## Abstract

Parental burnout is a specific syndrome resulting from enduring exposure to chronic parenting stress. But why do some parents burn out while others, facing the same stressors, do not? The main aim of this paper was to propose *a theory of parental burnout* capable of *predicting* who is at risk of burnout, *explaining* why a particular parent burned out and why at that specific point in time, and *providing directions* for intervention. The secondary goal was to operationalize this theory in a tool that would be easy to use for both researchers and clinicians. The results of this two-wave longitudinal study conducted on 923 parents suggest that the Balance between Risks and Resources (BR^2^) theory proposed here is a relevant framework to predict and explain parental burnout. More specifically, the results show that (1) the BR^2^ instrument reliably measures parents' balance between risks (parental stress-enhancing factors) and resources (parental stress-alleviating factors), (2) there is a strong linear relationship between BR^2^ score and parental burnout, (3) parental burnout results from a chronic imbalance of risks over resources, (4) BR^2^ predicts parental burnout better than job burnout and (5) among the risk and resource factors measured in BR^2^, risks and resources non-specific to parenting (e.g., low stress-management abilities, perfectionism) equally predict parental and job burnout, while risks and resources specific to parenting (e.g., childrearing practices, coparenting) uniquely predict parental burnout.

## Introduction

Every parent is familiar with the curious paradox that parenting is at the same time one of the most energy-consuming and one of the most energy-giving activities. It both taxes and replenishes your emotional resources. It empties you and nourishes you. Fortunately, the positive aspects of parenting usually compensate for—and even outweigh—the stressful and negative aspects. Thus, for most parents, the balance between resources and demands is either positive or in equilibrium. But what happens when the balance leans chronically to the wrong side? As we will show in this paper, that is precisely when parents are at risk of parental burnout.

### Parental burnout

Parental burnout is a unique and context-specific syndrome resulting from enduring exposure to chronic parenting stress (Roskam et al., 2017; Mikolajczak et al., 2018b). The first and main symptom is an overwhelming exhaustion related to one's parental role: parents feel tired when getting up in the morning and having to face another day with their children; they feel emotionally drained by the parental role to the extent that thinking about their role as parents makes them feel they have reached the end of their tether. A second symptom is an emotional distancing from their children: exhausted parents become less and less involved in parenting and the relationship with their children; interactions are limited to functional/instrumental aspects at the expense of emotional aspects. A third symptom is a loss of accomplishment in one's parental role: parents feel fed up with parenting, they cannot stand their role as father/mother anymore, and they no longer enjoy being with their children. Importantly, all these symptoms and states contrast with both how the parent felt before about parenting (Roskam et al., 2018).

Parental burnout seems to have far-reaching consequences for the families concerned. A recent study conducted on 1,551 parents (Mikolajczak et al., 2018a) shows that parental burnout has detrimental consequences for the parent (escape ideation and suicidal thoughts, increase in addictive behavior, sleep disorders, health disorders), for the couple (increase in the frequency and intensity of conflicts), and for the child(ren) [neglectful and violent behavior toward the child(ren)]. While the effect of parental burnout on the parent him/herself is comparable in size to that of job burnout, its effect on neglectful and violent behavior toward the child(ren) is much larger than that of job burnout. As a matter of fact, when their co-variation is controlled for, parental burnout explains 31% of the frequency of neglectful and violent behavior toward children, while job burnout explains <1%. These consequences highlight the pressing need to develop targeted and efficient interventions to treat and prevent parental burnout.

### A theoretical framework for parental burnout

Treating and preventing parental burnout require to first understand it. What is needed is *a theory of parental burnout* capable of *explaining*^1^ why a particular parent burned out and why at that specific point in time and *predicting*^2^ who is at risk for parental burnout. In order to be really useful, this theory should have two additional features. First it needs to be operationalizable in a tool that will be easy to use for both researchers and clinicians. Second, it should provide clear directions for intervention.

**Figure 1:**
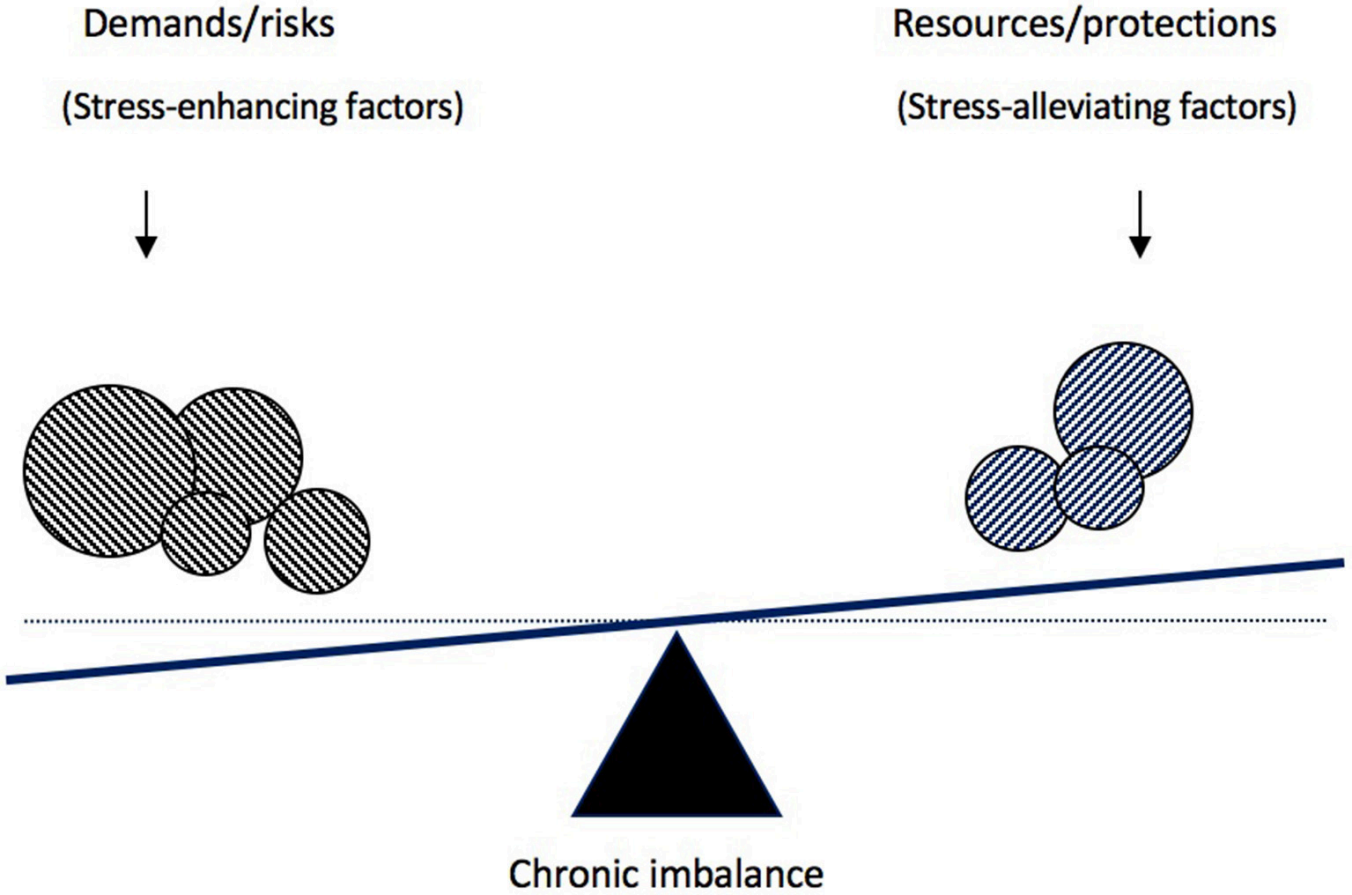
Schematic representation of the balance between risks and resources theory of parental burnout.

The notion of balance mentioned previously can be used to lay the foundation for such a theory. In organizational psychology, several models have proposed that the well-being of an individual at work is the result of the balance between demands on one side and resources on the other^3^. The most popular model is the Job Demand-Resources (JD-R) model (Demerouti et al., 2001; Bakker and Demerouti, 2007) which posits that “job burnout develops when job demands are high and when job resources are limited, because such negative working conditions lead to energy depletion and undermine employees' motivation, respectively” (Demerouti et al., 2001, p. 499). This model constitutes the best theory of job burnout so far, because it not only explains and predicts the occurrence of job burnout (Hakanen et al., 2008) but also provides clear directions for reducing burnout in organizations (see e.g., Schaufeli, 2017). In this paper, we will import the central tenets of the JD-R into the parenting domain. We will also refine the theory and its operationalization. Indeed, a simple transposition of the JD-R to parenting would lead to the conclusion that “parental burnout develops when parental demands are high and when parental resources are limited.” We propose instead that “parental burnout develops when parental resources are insufficient to meet the demands (whatever they are).” In other words, we suggest that parental burnout results from a chronic imbalance of demands (risk factors) over resources (protection factors) (see Figure 1).

Given that burnout is a stress-related disorder, we define demands/risk factors as factors that significantly *increase* parental stress. Examples of such stress-increasing factors are parental perfectionism, low emotional intelligence, poor childrearing practices, countless parental duties and chores, lack of support from the co-parent, lack of external support (family support, nurseries, etc.). By contrast, resources/protection factors can be defined as factors that help to significantly *decrease* parental stress. Examples of such stress-alleviating factors are parental self-compassion, high emotional intelligence, good childrearing practices, time for leisure, positive coparenting, external support, etc. As these examples clearly show, resources are not the absence of risks, but the opposite of risks. In other words, the mere fact that you do not spend stressful time with your children does not mean that you spend quality/resourcing time with them; the mere fact that your coparent does not denigrate you in your parental role does not mean that s/he values you; and the mere fact that you do not lack money does not mean that you have enough money to afford a full-time domestic helper to alleviate your parental chores. This theoretical clarification is important for the operationalization of the balance that will follow.

Another point that will be important for the operationalization of the balance is that although risk and resource factors can theoretically belong to any of the five levels of Bronfenbrenner (1979) (viz. individual, microsystem, mesosystem, exosystem and macrosystem), factors at the exosystem and macrosystem levels are distal factors that often increase (or decrease) parental stress *through* their impact on parents' duties and cognitions. For instance, nurseries decrease parental stress because they alleviate parental chores; state recommendations about childrearing (e.g., five fruits and vegetables a day, no television and no videogames before the age of six, warm and positive parenting) increase parental stress only if they are adopted by parents and raise their parental standards. It therefore follows that it may not be necessary to assess risk and resource factors at the exosystem and macrosystem levels if we can find a way to assess their impact on the parent's life.

### Operationalizing the balance between risks and resources theory (BR^2^)

**Figure 2:**
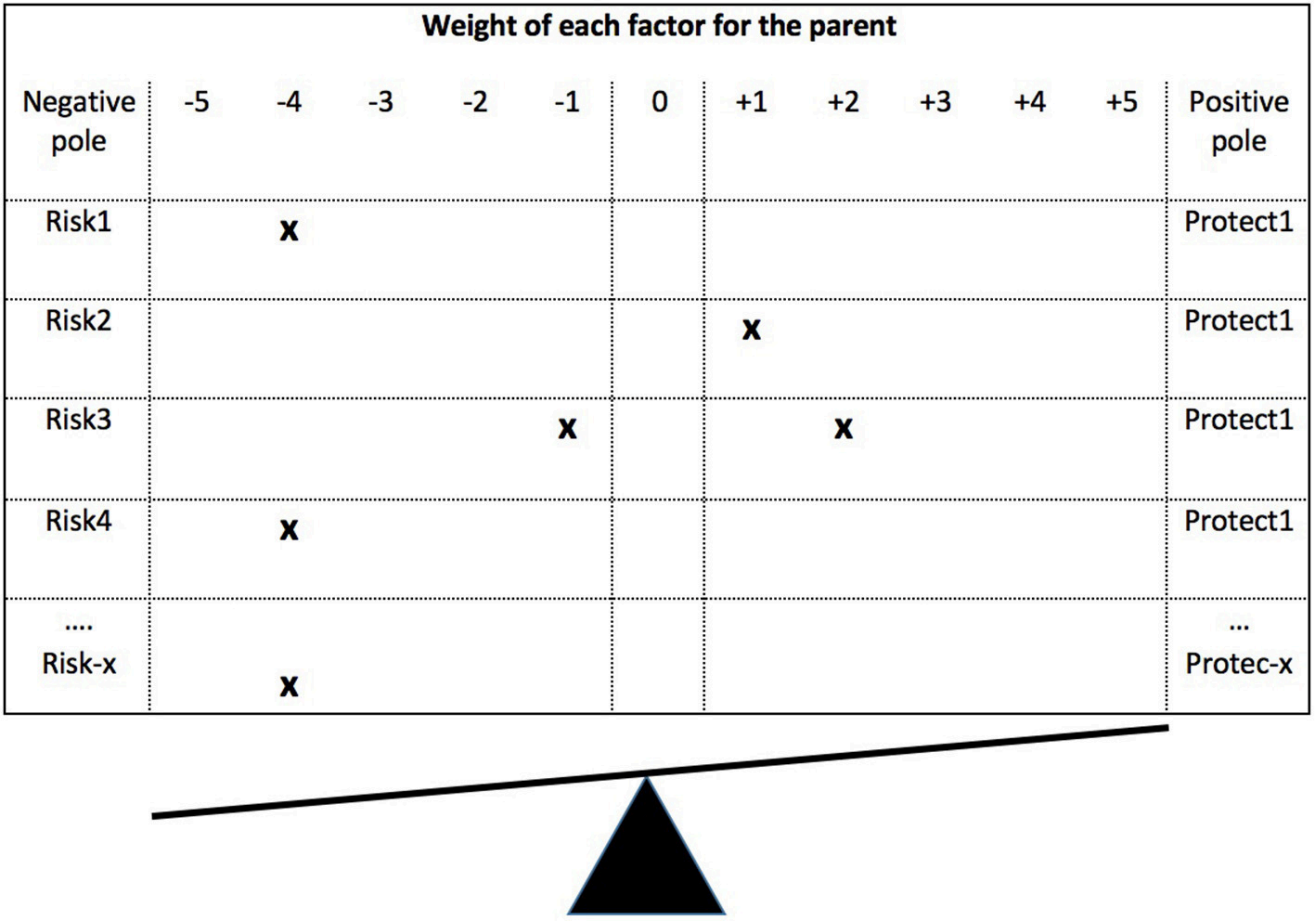
Operationalization method of the balance between risks and resources.

Efficiently operationalizing the Balance between Risks and Resources Theory implies choosing the right format and the right content. As regards the *format*, we sought a format that would reflect the very notion of balance. Thus, instead of developing several items to measure risks and other items to measure resources and treating them separately (as is currently done in the organizational domain), we took advantage of the fact that resources are the opposite of risks in order to develop bipolar items, in which the left pole is the risk factor and the right pole the protection (e.g., Left pole - Risk: My partner denigrates me as a mother/father. Right pole - Resource: My partner says that I am a good mother/father). The response scale goes from−5 (full endorsement of the risk factor) to +5 (full endorsement of the protection factor), 0 indicating that the parent has neither the risk factor nor the protection factor (in this case: my partner does not denigrate me but does not explicitly value me either). The principle is illustrated in Figure 2. Provided that the questionnaire includes the most important risk/resource factors and that these are appropriately weighted (e.g., heavier risks/protections reflected by more items; see below), the arithmetic sum of the answers to the questionnaire^4^ should reflect the parental balance between risks and protections. If the parent has more or heavier risk factors, the score will be negative; if protections just compensate for risks, the score will be zero. If the parent has more (or heavier) protection factors, the score will be positive.

As regards the *content* of the balance (i.e., the items), deciding which factors should be included requires prior knowledge of the factors that may constitute significant risks or protections vis-à-vis parental burnout. Information about the weight of each risk/protection factor is also needed because, obviously, we cannot expect a small protection to compensate for a big risk. We summarize in Appendix A the available knowledge about risk/protection factors potentially applicable to a majority of parents and about the strength of their respective association with parental burnout. In order to make the instrument widely applicable, we excluded from the Table—and from the instrument– risk/protection factors that would be only applicable to a minority of parents (for example, parents of ill or disabled children; see e.g., Lindström et al., 2011; Basaran et al., 2013). Based on this information, we included in the balance factors that had *at least* a weak association with parental burnout and excluded all others (as will be shown in the Results section and as expected, including those factors did not increase the explanatory power of the balance). We added to these factors two factors that were not investigated in previous quantitative studies but which emerged as highly relevant in both qualitative studies and from our clinical experience with burned-out parents: parental load (too many demands, too many things to do and not enough time to do them) and views on child-rearing divergent from those of the other people who take care of the child (nursery staff, teachers, grandparents, etc.).

We also used information provided by previous studies and clinical experience to weight the factors: factors with the strongest association with parental burnout would need to be given more weight than factors with weaker associations. Instead of a complicated weighting system (e.g., a system by which each item would have to be multiplied by a given coefficient), we opted for a simpler system: factors strongly associated with parental burnout would be represented by more items than factors weakly associated with it (which has the additional advantage of increasing the measurement reliability of the most important factors). Strong predictors were represented by approximately four bipolar items, moderate ones by three bipolar items and weak ones by one bipolar item^5^. In order to avoid giving excessive weight to highly correlated factors, we reduced this number where necessary in order to take account of the interdependence between predictors (see Table 2 in Mikolajczak et al., 2018b). Items were written, based on the literature, by two university professors and one Ph.D. student, and discussed until consensus was reached.

### Overview of the present study

The goal of this study was to test the theoretical assumption that parental burnout results from an imbalance between parental risks and protection factors and to propose a way to efficiently operationalize this theory. The first part of the paper will be devoted to the operationalization of the balance between risks and resources (hereafter named BR^2^). We describe how we compared the predictions made with four versions of BR^2^ (without or with demographic factors—with several ways to take them into account) in order to come up with the most parsimonious AND valid measure possible. In the second part of the paper, we examine more closely the hypothesis that parental burnout results from an *im*balance between parental risks and protection factors. Validating the theory that parental burnout results from an *im*balance between parental risks and resources involves first showing that BR^2^ scores largely predict parental burnout scores and then showing that a *negative imbalance* leads to parental burnout. If this is confirmed, a last check would need to be made before considering that the Balance between parental Risks and Resources offers an interesting theoretical framework for *parental* burnout: showing that BR^2^ score predict parental burnout better than it predicts job burnout. Even more specifically, it would be necessary to show that *common risk factors* (e.g., a perfectionist personality, poor stress management abilities, pessimistic tendencies) contribute equally to the prediction of parental and job burnout, while *specific risk factors* inherent to the parental domain (e.g., high parental standards, poor parenting practices, poor co-parenting) predict only parental burnout.

## Methods

### Participants

Data were collected from a sample of 923 French-speaking parents who had at least one child living at home. They participated in a two-wave longitudinal study conducted in May and November 2017. The participants were 728 women (78.9%) and 195 men (21.1%). The women's ages ranged from 22 to 64 (mean age = 39.39; SD = 7.14), and the men's ages ranged from 27 to 69 (mean age = 43.07; SD = 9.53). The majority of the sample came from Belgium (97%), a minority from other French-speaking European countries (2.3%) and the remaining 0.7% from non-European French-speaking countries. 51.1% of participants were married, 30.4% were legal cohabitants and 18.4% were single parents. Overall, the participants had from 1 to 7 children, aged from 0 to 35 years old (mean age = 8.97; SD = 6.80). 13.8% of participants were educated to secondary level, 37.8% had a first degree from university or college, and 48.4% had a master's degree, a Ph.D. or MBA degree. Income was distributed as follows: 20.4% of the sample had a net monthly household income lower than €2,500, 44.3% between €2,500 and €4,000, 25.2% between €4,000 and €5,500, and 10.1% higher than €5,500.

### Procedure

The current study received the approval of the Institutional Review Board. Participants were informed about the survey through social networks, websites, schools, pediatricians or word of mouth. In order to avoid (self-)selection bias, participants were not informed that the study was about parental burnout. The study was presented as a study about parental well-being and exhaustion. Parents were eligible to participate in the study only if they had (at least) one child still living at home. Participants were invited to complete the survey after giving informed consent. The informed consent they signed allowed participants to withdraw at any stage without having to justify their withdrawal. They were also assured that data would remain anonymous. At the end of the first wave assessment (Time 1), parents were asked if they would agree to be contacted 6 months later to participate in the second wave of data collection (Time 2). Of the 4,390 parents who clicked on the survey link, 1,551 parents fully completed the survey at Time 1 and 923 of them (59.51%) fully completed it at Time 2. Time-invariant socio-demographic variables, e.g., sex, were only collected at Time 1. Time-varying socio-demographic variables, e.g., number of children, were collected at Times 1 and 2. The balance between risks and resources was collected at Time 1, while parental as well as job burnout were both collected at Times 1 and 2.

Participants who completed the questionnaire at Time 1 had the opportunity to enter a lottery with a chance of winning €300, a stay for two persons in a hotel, or amusement park or spa tickets. Those who participated at Time 2 had the opportunity to enter a lottery with a chance of winning €300, €200, or amusement park tickets. Participants who wished to participate in the lottery and/or to participate in the second wave of data collection had to provide their email address, but the latter was disconnected from their questionnaire. The questionnaire was completed online with the forced choice option on, ensuring a dataset with no missing data.

### Measures

Parental burnout was assessed with the Parental Burnout Inventory^6^ (PBI, Roskam et al., 2017), a 22-item self-report questionnaire created on the basis of a deductive approach starting from the tridimensional model of professional burnout (Maslach and Jackson, 1981; Maslach et al., 2001). The PBI consists of three subscales: Emotional Exhaustion (8 items) (e.g., “I feel emotionally drained by my parental role”), Emotional Distancing (8 items) (e.g., “I sometimes feel as though I am taking care of my children on autopilot”), and Loss of Parental Accomplishment (6 items) [e.g., “I accomplish many worthwhile things as a parent” (reversed)]. Items are rated on 7-point Likert scales: “never” (0), “a few times a year or less” (1), “once a month or less” (2), “a few times a month” (3), “once a week” (4), “a few times a week” (5), “every day” (6). In the current sample, Cronbach's alphas were 0.92, 0.89, 0.85 for the three subscales and 0.91 for the total score.

Job burnout was assessed with the Maslach Burnout Inventory-General Survey (MBI-GS; Schaufeli et al., 1986, 1996). The MBI is a widely used 16-item questionnaire encompassing three factors, i.e., emotional exhaustion (5 items), cynicism (5 items) and professional efficacy (6 items). Items are in the form of “I feel emotionally drained from my work.” The instruction is as follows: “Please read each statement carefully and decide if you ever feel this way about your job.” Likert-type scales are in the form of “How often?,” with a 7-point scale of frequency, i.e., “never” (0), “a few times a year or less” (1), “once a month or less” (2), “a few times a month” (3), “once a week” (4), “a few times a week” (5), “every day” (6). The global score was computed after reversing the items of the professional efficacy factor, so that higher scores indicated greater burnout. The Cronbach alpha found in the current sample was 0.82.

#### Socio-demographic factors

Participants were asked about their age, sex, number of children (plus the age of each child and whether s/he was still living at home), marital status, level of education, net household income, and work regimen.

The Balance between Risks and Resources (BR^2^) was assessed by means of 39 bipolar rating scales encompassing 11 levels, i.e., from −5 to +5 going through 0. The negative pole represented the risk while the positive pole represented the corresponding resource. For example, −5: “My partner denigrates me as a mother/father;” +5: “My partner says that I am a good mother/father.” The global score was computed by summing the 39 items so that positive scores indicated that the parent had more (or heavier) resources than risks, negative scores indicated that the parent had more (or heavier) risks than resources, and zero scores indicated that the parent had the same level of risks and resources. The BR^2^ items are presented in both English and French in Appendix B and C.

Social desirability was assessed using the short form of the Marlowe-Crowne social desirability scale (Reynolds, 1982) in order to control for socially desirable responses. The scale is composed of 12 items rated on a true-false response scale. The items are in the form of “*I'm always willing to admit when I make a mistake.”* Over the 12 items, seven were reversed so that the true response corresponded to high desirability. For example, the item “*I sometimes feel resentful when I don't get my way*” was reversed. The 0 (no desirability)−1 (desirability) scores were summed across the 12 items. The internal consistency (Cronbach's alpha) was 0.61.

### Data analyses

As a preliminary analysis, we examined drop-out at Time 2. Attrition is usual in longitudinal research. One-way ANOVAs were conducted for continuous and ordinal variables and χ2 for categorical ones, to test whether there were significant differences between the participants who dropped out and the others with regard to socio-demographic data, parental burnout, and BR^2^ at Time 1.

In order to test whether the socio-demographic risks and resources should be considered in the BR^2^ instrument, the BR^2^ score was computed using three different methods for comparison purposes. In the first method (BR^2^), the 39 BR^2^ items were summed without considering the socio-demographic factors. The score theoretically ranged between −195 and +195. In the second and third methods, sociodemographics were included in the balance. In order to determine which sociodemographic risks and resources had to be included, we started by testing the relations between parental burnout and the sociodemographic factors, i.e., age and sex of the parent, number of children still living at home, mean age of the children, marital status, educational level, incomes, and work regimen. For the continuous variables (i.e., age of the parent and age of the children), Spearman correlations with the parental burnout score were computed. Because the correlation coefficients were negligible (0.008 for parent's age and 0.02 for children's mean age), age variables were no longer considered in the computation scores of the following BR^2^ scores. For the categorical (e.g., sex) and ordinal (e.g., educational level) variables, the nature of each sociodemographic risk and its corresponding resource was defined according to the result of mean comparisons and post-hoc tests used to identify which subgroups of parents were most at risk of parental burnout. For instance, analyses revealed that for the variable “marital status,” single parents were more at risk of parental burnout than those in the married or legal cohabitant categories. The results of mean comparisons and post-hoc tests are presented in Table 1. Since it is possible that small, statistically non-significant, risk/resource factors have an effect when combined with others, the six sociodemographic factors, i.e., sex, number of children still living at home, marital status, educational level, incomes, and work regimen, were converted into bipolar items and added to the 39 regular BR^2^ items for the computation of the second and third BR^2^ scores, regardless of whether the mean comparisons were statistically significant or not. In the end, the sociodemographic risks were: being a woman, having at least three children still living at home, being a single parent, having a university degree, having a net monthly household income lower than €2,500, and working part-time or having no professional occupation. In the second BR^2^ score (BR^2^ with sociodemographics_−1/0/+1), a −1/0/+1 rating scale was applied to these seven sociodemographic items, so that the total BR^2^ score ranged between −202 and 202. For example, single parents were coded −1, while married and cohabitant parents were coded +1 on the marital status item. In the third BR^2^ score (BR^2^ with sociodemographics_−5/0/+5), a −5/0/+5 rating scale was applied to these seven sociodemographic items, so that the BR^2^ score ranged between −230 and 230. For example, single parents were coded −5, while married and cohabitant parents were coded +5 on the marital status item. We then tested bivariate associations among the variables included in the study at Times 1 and 2 for replication purposes, i.e., parental and job burnout, and for the three BR^2^ scores. In order to select the best BR^2^ model, i.e., including socio-demographics or not, correlation coefficients between parental burnout on the one hand and the three BR^2^ scores on the other hand were compared.

**Table 1:** Means (Standard Deviations) and Mean Comparisons of Parental Burnout according to Sex, Number of Children Living at Home, Marital Status, Educational Level, Income, and Work Regimen.

**Sex**		**Number of children**			**Marital status**		**Educational level**			**Income**			**Work regimen**		
**Women**	**Men**	**1**	**2**	**≥3**	**Married or legal cohabitant**	**Single parenthood**	**College**	**High School**	**University**	**<2500€**	**2500-4000€**	**>4000€**	**Full-time**	**Part-time**	**No job**
34.68 (23.01)	29.47 (18.65)	28.31 (20.30)	36.01 (22.42)	37.92 (23.41)	33.27 (21.58)	34.97 (25.02)	33.51 (22.28)	32.62 (22.10)	34.34 (22.39)	34.96 (23.64)	33.57 (22.77)	32.79 (20.76)	31.63 (21.43)	35.76 (22.77)	37.47 (23.92)
*F*_(1, 922)_ = 8.48^**^		*F*_(2, 920)_ = 16.39^***^			*F*_(1, 921)_ = 0.81		*F*_(2, 920)_ = 0.59			*F*_(2, 920)_ = 0.56			*F*_(2, 920)_ = 5.18^**^		

** *p < 0.01*;

*** *p < 0.001*.

To test the relationships between the resulting version of BR^2^ and parental burnout both cross-sectionally and longitudinally, BR^2^ at Time 1 was regressed on parental burnout at Times 1 and 2. The linear relationships were illustrated graphically in order to check the core assumption that parental burnout occurs when risks exceed resources in the balance (negative scores in BR^2^).

To test the specificity of BR^2^ vis-à-vis parental burnout in comparison to job burnout, we ran correlations between BR^2^ and parental and job burnout respectively, and we compared the coefficients. In order to go deeper into this issue, we split the BR^2^ score into two subscores. The first one was the sum of the BR^2^ items measuring *common antecedents*, i.e., risks and resources that were expected to have a similar influence on parental and job burnout, for example perfectionism or ability to manage stress. The second one was the sum of the BR^2^ items measuring *specific antecedents*, i.e., risks and resources that were expected to have a greater influence on parental burnout than on job burnout, for example parenting styles or co-parenting. The allocation to common or specific antecedent categories is given in the first column of Appendix B for each of the 39 items. We then applied hierarchical multiple regression analyses to estimate the incremental validity of specific antecedents (second BR^2^ subscore) in predicting parental burnout over and above common antecedents (first BR^2^ subscore). The same was done with job burnout as the dependent variable. Values of tolerance (2.03) and VIF (.19) support the absence of collinearity between the two intermediate BR^2^ scores. The analyses were conducted twice, i.e., at Times 1 and 2, for replication purposes.

## Results

### Preliminary analyses

There were no significant differences between parents who completed the survey at Time 1 and Time 2 and those who dropped out with regard to gender, age, number of children, marital status, work regimen, parental burnout and BR^2^ at Time 1. The drop-out subjects were only found to be slightly less educated than the other participants and their net monthly household income was also slightly lower than that of the other participants.

### Should sociodemographic factors be included in BR2?

The correlations between the three BR^2^ scores, i.e. the basic score and the two scores considering six additional sociodemographic items (either in the form −1/0/+1 or in the form−5/0/+5), and parental burnout at Times 1 and 2, are presented in Table 2. Correlations involving the basic score and the two alternative ones were similar at Time 1 and at Time 2. These results indicated that considering sociodemographic factors in the BR^2^ instrument did not improve the model or its predictive power. The BR^2^ basic score, consisting of the sum of the 39 regular items presented in the Appendix B (in English) and C (in French), was therefore considered the most relevant and the most parsimonious instrument. Therefore, sociodemographic antecedents were no longer considered in subsequent analyses.

**Table 2:** Correlations between BR^2^, BR^2^ with Sociodemographics, Parental Burnout, and Job Burnout at Time 1 and Time 2.

	**Parental burnout_Time1**	**Parental burnout_Time 2**	**Job burnout_Time 1**	**Job burnout_Time 2**
BR^2^	−0.58	−0.53	−0.40	−0.36
BR^2^ with sociodemographics_1	−0.58	−0.53	−0.40	−0.36
BR^2^ with sociodemographics_2	−0.58	−0.52	−0.40	−0.35
Parental burnout_Time 1	–	0.77	0.36	0.30
Parental burnout_Time 2	–	–	0.33	0.40
Job burnout_Time 1	–	–	–	0.70

*BR^2^ with sociodemographics_1 includes the 6 relevant sociodemographic antecedents coded −1/+1; BR^2^ with sociodemographics_1 includes the 6 relevant sociodemographic antecedents coded −5/+5; All correlations are significant at p < 0.001*.

### Relationship between BR^2^ and parental burnout

**Figure 3:**
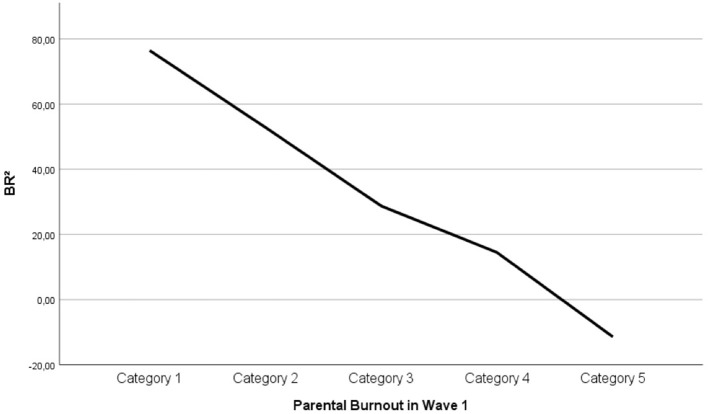
Linear relation between BR^2^ and parental burnout at Wave 1. Category 1: 2/3 of the symptoms never to a few times a year; Category 2: once a month or less; Category 3: a few times a month; Category 4: a few times a week; Category 5: everyday.

**Figure 4:**
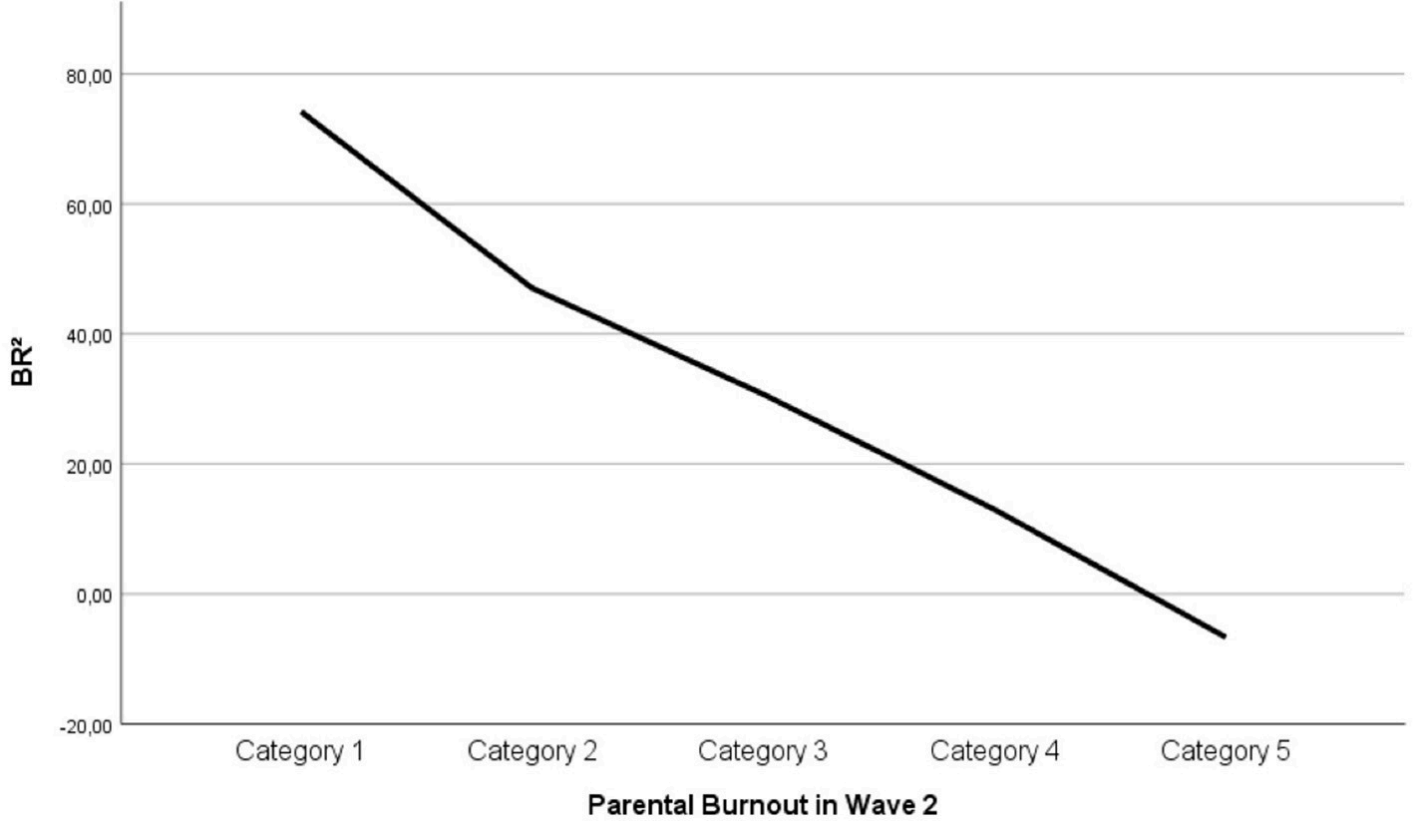
Linear relation between BR^2^ and parental burnout at Wave 2. Category 1: 2/3 of the symptoms never to a few times a year; Category 2: once a month or less; Category 3: a few times a month; Category 4: a few times a week; Category 5: everyday.

In the linear regression model, BR^2^ was found to predict parental burnout both cross-sectionally, β = −0.58, *p* < 0.001, and prospectively, β = −0.53, *p* < 0.001. The amount of variance of parental burnout (R2) explained by BR^2^ was 34% cross-sectionally and 28% prospectively. The linear relations between BR^2^ and parental burnout are displayed in Figures 3, 4. They illustrate that, as predicted, parents whose balance leans to the negative side can be considered to be in burnout (they display at least 2/3 of the symptoms every day^7^). The same pattern was replicated at Times 1 and 2. All correlations hold when social desirability is controlled for. The size of correlations computed with or without control for social desirability does not statistically differ.

### Specificity of BR^2^ vis-à-vis parental burnout

The correlations between BR^2^ and both parental and job burnout at Time 1 and Time 2 are displayed in Table 2. The comparisons between the correlations involving either parental or job burnout showed that BR^2^ was significantly more closely related to parental burnout than to job burnout both at Time 1, Z = −5.12, *p* < 0.001, and at Time 2, Z = −4.44, *p* < 0.001.

The next step aimed to check the incremental validity of specific antecedents (second BR^2^ subscore) in predicting parental burnout over and above common antecedents (first BR^2^ subscore), under the hypotheses that the variance explained by common antecedents is the same for parental and job burnouts and that the incremental value of specific antecedents would be high for parental burnout but low for job burnout. As expected and as shown in the first step of the hierarchical linear analyses presented in Table 3, common antecedents were similarly related to both parental and job burnout, with a total of 22% (Time 1) and 20% (Time 2) for parental burnout and 20% (Time 1) and 14% (Time 2) for job burnout. As expected and as shown in the second step, specific antecedents were only related to parental burnout, with an additional amount of variance explained of 12% at Time 1 (ΔR2 = 0.12, *p* < 0.001) and 8% at Time 2 (ΔR2 = 0.08, *p* < 0.001).

**Table 3:** Hierarchical multiple regression analyses predicting parental and job burnout at time 1 and 2 from common and specific BR^2^ antecedents.

	**Parental burnout_Wave 1**		**Parental burnout_Wave 2**		**Job burnout_Wave 1**		**Job burnout_Wave 2**	
	**Δ*R2***	**β**	**Δ*R2***	**β**	**Δ*R2***	**β**	**Δ*R2***	**β**
Step 1	0.22^**^		0.20^**^		0.20^**^		0.14^**^	
BR^2^_common antecedents		−0.47^**^		−0.45^**^		−0.44 ^**^		−0.38^**^
Step 2	0.12^**^		0.08^**^		0.00		0.00	
BR^2^_common antecedents		−0.12^**^		−0.17^**^		−0.43^**^		−0.34^**^
BR^2^_specific antecedents		−0.50^**^		−0.39^**^		−0.02		−0.06
Total *R*^2^	0.34^**^		0.28^**^		0.20^**^		0.14^**^	

** *p ≤ 0.001*.

## Discussion

This paper aimed to provide a theoretical framework for parental burnout that would be widely applicable, easily operationalizable and accurate in its predictions. The findings suggest that the Balance between parental Risks and Resources theory and its operationalization vehicle BR^2^ meet these criteria. As predicted, parental burnout scores are a linear function of the Balance between Risks and Resources (BR^2^). The large size of the correlations suggest that the most important risk and resource factors are included in the balance. Adding socio-demographic factors did not increase the percentage of explained variance. This is not surprising, because demographic factors (e.g., having a disabled or chronically ill child) lose their effect when other factors such as parental load, leisure time, or support are taken into consideration (while the opposite is not true). Even though there is unexplained variance left in the model, which suggest that a number of risks and resources could be added (see limitation section), the current operationalization of BR^2^ fully supports the theory that burnout is the result of an imbalance between risk and resources: as soon as the balance leans on the negative side (i.e., risks outweigh resources), the parent starts experiencing most burnout symptoms every day (s/he can be said to be “in burnout”).

In accordance with the old adage that there is nothing more practical than a good theory, BR^2^ simultaneously provides a *theoretical framework* that explains why a given parent has burned out and a *practical tool* for the easy identification of the factors that should be targeted to reduce (the risk of) parental burnout. If the balance points to several risk factors, the arithmetic principle of the balance suggests that preference should be given to risk factors that have the greatest weight (i.e., those that are closest to −5), provided that they are controllable in the parent's situation. This is not always the case. For instance, poor coparenting is a weighty factor, but if the coparent does not want to change or get involved, other factors will need to be targeted instead. The very principle of the balance implies that reducing several smaller risks should be equivalent to reducing one big one, and that if a risk cannot be removed, adding sufficient resources should cancel its impact. These are amongst the strongest clinical implications of the theory, which will need to be confirmed through intervention studies. It would also be interesting to investigate if individual/group psychotherapy programs for parents or parental empowerment programs (Tremolada et al., 2011) could help restore an equilibrium.

Another result that has important clinical implications is that, as expected, *common risk factors* (e.g., a perfectionist personality, poor stress management abilities, pessimistic tendencies) contributed equally to the prediction of parental and job burnout, while *specific risk factors* inherent to the parental domain (e.g., high parental standards, poor parenting practices, poor co-parenting) predicted only parental burnout. This suggests that parents who have mainly *common risk factors* in their balance will be vulnerable to both forms of burnout, while parents who have mainly *specific risk factors* inherent to the parental domain in their balance will be vulnerable only to parental burnout. This implies that if a parent (who also has a job) has two equally weighty factors in his or her balance, one of which is non-specific while the other is specific, the non-specific factor should be targeted first. Intervention studies with long-term follow-ups of both parental and job burnout are needed to confirm this.

As these examples show, this study opens several directions for future research. In addition to testing the predictions stemming from the theory and refining it accordingly, more work needs to be done on the BR^2^ instrument. Indeed, one of the limitations of this study is that it is based on Western research and conducted in a Western country. While BR^2^ *theory* is equally applicable to different environments and cultures (because the theory can accommodate risks and resources other than those mentioned here), the current version of the BR^2^ *instrument* may be more suitable, i.e., accurate in its predictions, for typical Western parents. Indeed, it does not contain risk/resource factors that would only be relevant to specific cultures (e.g., Japanese women's difficulties in restarting a career after having their first child). The BR^2^ *instrument* will therefore need to be adapted for use in other cultures. Moreover, it does not contain very specific risk/resource factors that would only apply to only a minority of parents (e.g., the constant financial concerns that very disadvantaged parents may have) or to very specific contexts; therefore, some risk/resource factors may need to be added or removed and/or the weight of some factors may need to be changed (e.g., pressure to be a perfect parent may have more weight amongst parents who are the subject of a priori negative social judgments of their parenting abilities, such as blind or same-sex parents).

This being said, the BR^2^ *instrument* may need refinements even for use with typical Western parents. Indeed, another limitation of this study is that the factors included in the balance and their individual weight were defined on the basis of a small number of available studies. The results of several other studies are expected soon (Cf. special issue in Frontiers: “When the Great Adventure of Parenting Turns to Disaster: Regrets and Burnout) and other studies are sure to follow. Their findings may lead to the addition of new factors and/or to changes to the weighting of the factors already included in the balance. It is our hope that the BR^2^ *instrument* will continue to evolve on the basis of future research findings in order to further increase its explanatory power and its usefulness to clinicians working with exhausted parents.

## Concluding comment

The BR^2^ theory and instrument presented in this paper suggest that parental burnout threatens any parent who accumulates too many risks without enough compensatory resources. The nature of risks and resources is peculiar to each parent (thus each burnout has its own etiology/history), but the underlying process (imbalance between risks and resources) seems common to all burned-out parents. In addition to its interest for researchers, the BR^2^ model is a simple model to understand, represent and use for clinicians and parents. The metaphor of the balance makes it particularly accessible and functional: any parent can easily imagine what is weighing heavily on one side and what lightens the load on the other side.

## Ethics statement

This study was carried out in accordance with the recommendations and the ethical standards of the institutional research committee and with the 1964 Helsinki Declaration and its later amendments. The protocol was approved by the Commission éthique de l'Institut de Recherches en Sciences Psychologiques de l'Université catholique de Louvain. All subjects gave written informed consent in accordance with the Declaration of Helsinki.

## Author contributions

IR devised the theoretical framework of the balance in the framework of a discussion with MM. The authors worked together to operationalize it, design the study, and collect the data. IR analyzed the data and wrote the Methods and Results section. MM wrote the Introduction and Discussion sections. Both authors commented on, proofread and approved the final version of the paper.

### Conflict of interest statement

The authors declare that the research was conducted in the absence of any commercial or financial relationships that could be construed as a potential conflict of interest.

## Footnotes

^1^For intervention purposes.

^2^For prevention purposes.

^3^The first such model was the Demand-Control model of Karasek (1979), followed by the Effort-Reward Imbalance model of Siegrist et al. (1986) and, more recently the Job Demand-Resources (JD-R) model (Demerouti et al., 2001).

^4^This arithmetic sum logically involves subtracting risks from resources.

^5^In order to reflect the fact that strong predictors have correlations with parental burnout of around 0.40, moderate predictors of around 0.30 and weak ones of around 0.10.

^6^Items EE1 to EE8 and PA1 to PA6 copyright © 1981 Christina Maslach and Susan E. Jackson. All rights reserved in all media. Published by Mind Garden, Inc.,www.mindgarden.com. Altered with permission of the publisher.

^7^Until studies are conducted that make it possible to formally determine the clinical cut-offs at the PBI, this criterion was adopted. It is even more restrictive than that used for determining whether a person is in a state of job burnout or depression (according to the DSM, people need to present 55% of the symptoms of depression every day to be considered “clinically depressed”).
