# Supplementary material for: A Theoretical and Clinical Framework for Parental Burnout: The Balance Between Risks and Resources (BR2)
Source: Front Psychol. 2018 Jun 12;9:886. doi: 10.3389/fpsyg.2018.00886 (PMC6006266; doi:10.3389/fpsyg.2018.00886)
Supplement: Supplementary file 1 [file Data_Sheet_1.docx]

**Appendices A, B and C of the manuscript**

A theoretical and clinical framework for parental burnout:

The Balance between Risks and Resources (BR²)

Moïra Mikolajczak and Isabele Roskam

Université catholique de Louvain, Louvain-la-Neuve, Belgium

**Appendix A**

**Summary of the risk and resource factors vis-à-vis parental burnout and their individual weight (based on data available when the balance was developed)**

| **Category** | **Risk/resource factor** | **Studies** | **N (for each study)** | **Effect-size (r)** | **Aggregated effect size r (after Fisher to z transformation)^b^** | **Interpretation of overall effect ^c^** |
| --- | --- | --- | --- | --- | --- | --- |
| Socio-demographics | Sex of the parent (for men = 1, women = 2) | Lindahl-Norberg, 2010; Lindström et al., 2011^a^; Mikolajczak et al., 2018 | N = 44 N = 251 N = 1723 | .31 .15 .06 | .08 | Trivial |
|  | Age of the parent | Mikolajczak et al., 2018 | N = 1723 | -.04 | -.04 | Trivial |
|  | Sex of the children | Lindström et al., 2011 ^a^; Mehauden & Piraux, 2018 | N = 251 N = 1723 | .01 .04 | .04 | Trivial |
|  | Age of the children | Lindström et al., 2011 ^a^; Mehauden & Piraux, 2018 | N = 251 N = 1723 | -.15 -.10 | -.11 | Small |
|  | Number of children | Mikolajczak et al., 2018 | N = 1723 | .04 | .04 | Trivial |
|  | Family structure (single parents vs biparental family) | Lindström et al., 2011 ^a^; Mikolajczak et al., 2018 | N = 251 N = 1723 | .09 .00 | .01 | Trivial |
|  | Family recomposition (“classical biparental” vs blended family) | Mikolajczak et al., 2018 | N = 1453 | .03 | .03 | Trivial |
|  | Work regimen (full-time, part-time, housewife/husband) | Lindström et al., 2011 ^a^; Mehauden & Piraux, 2018 | N = 251 N = 1646 | .22 .14 | .15 | Small |
|  | Level of education | Lindahl-Norberg, 2010; Lindström et al., 2011 ^a^; Mikolajczak et al., 2018 | N = 44 N = 251 N = 1723 | -.28 .00 .08 | .06 | Trivial |
|  | Net household income | Mikolajczak et al., 2018 | N = 1723 | .04 | .04 | Trivial |
|  | Perceived financial status | Lindahl-Norberg, 2010; Lindström et al., 2011 ^a^; Mehauden & Piraux, 2018 | N = 44 N = 251 N = 1723 | -.11 -.01 .00 | .00 | Trivial |
|  | House living area (including when controlling for number of children living at home) | Mikolajczak et al., 2018 | N = 1723 | -.01 | -.01 | Trivial |
|  | Characteristics of the neighborhood I (level of criminality) | Mehauden & Piraux, 2018 | N = 228 | -.05 | -.05 | Trivial |
|  | Characteristics of the neighborhood II (average income) | Mehauden & Piraux, 2018 | N = 228 | .12 | .12 | Small |
|  | Stress at work (for parents who hold a job) | Lindahl-Norberg, 2010; Piraux & Mehauden, 2018 | N = 44 N = 1557 | -.06 .06 | .06 | Trivial |
|  | Having a satisfying work/family/couple balance | Piraux & Mehauden, 2018; | N = 1719 | -.31 | -.31 | Medium |
|  | Having time for leisure | Piraux & Mehauden, 2018; Lindström et al., 2011^a^ | N = 1723 N = 251 | -.24 -.23 | -.24 | Medium |
| Life events | Loss of a family member | Piraux & Mehauden, 2018 | N = 1723 | .00 | .00 | Trivial |
| Psychological characteristics of the parent | Subjective self-construal (endorsement of Individualist-collectivist values) | Mehauden & Piraux, 2018 | N = 1723 | .10 | .10 | Small |
|  | Attachment anxiety | Mikolajczak et al., 2018 | N = 1723 | .29 | .29 | Medium |
|  | Attachment avoidance | Mikolajczak et al., 2018 | N = 1723 | .23 | .23 | Medium |
|  | Agreeableness | LeVigouroux-Nicolas et al., 2017 | N = 1723 | -.23 | -.23 | Medium |
|  | Neuroticism | LeVigouroux-Nicolas et al., 2017 | N = 1723 | .36 | .36 | Large |
|  | Emotional intelligence (including ability to express emotions and needs, to manage emotions and stress, to stand up for oneself and say no) | Mikolajczak et al., 2018 | N = 1723 | -.45 | -.45 | Large |
|  | Need for high degree of control | Lindström et al., 2011^a^ | N = 251 | .27 | .27 | Medium |
|  | Perfectionism/Performance-based self-esteem | Hubert & Aujoulat, under review; Lindström et al., 2011^a^; | N = 5 N = 251 | Strong (QS) .42 | .42 | Large |
|  | Parental self-efficacy beliefs | Mikolajczak et al., 2018 | N = 1723 | -.53 | -.53 | Large |
|  | Perceived role restriction | Mikolajczak et al., 2018 | N = 1723 | .40 | .40 | Large |
|  | Hyper-investment in one’s parental role | Gleis, 2018; Hubert & Aujoulat, under review | N = 5 N = 5 | Strong (QS) Strong (QS) | Strong (QS) | Large |
| Childrearing practices | Rules | Mikolajczak et al., 2018 | N = 1723 | -.17 | -.17 | Small |
|  | (In)consistent discipline | Mikolajczak et al., 2018 | N = 1723 | -.17 | -.17 | Small |
|  | Autonomy demands | Mikolajczak et al., 2018 | N = 1723 | -.22 | -.22 | Small to medium |
|  | Positive parenting | Mikolajczak et al., 2018 | N = 1723 | -.38 | -.38 | Large |
| Family functioning | Marital satisfaction | Lindström et al., 2011^a^; Mikolajczak et al., 2018 | N = 251 N = 1723 | -.27 -.42 | -.40 | Large |
|  | Agreeement between coparents | Mikolajczak et al., 2018 | N = 1723 | -.34 | -.34 | Medium to Large |
|  | Conflicts between coparents | Mikolajczak et al., 2018 | N = 1723 | .36 | .36 | Medium to Large |
|  | Support from the coparent | Lindström et al., 2011^a^; Mikolajczak et al., 2018 | N = 251 N = 1723 | -.21 -.29 | -.28 | Medium |
|  | Undermining attitude of the coparent | Mikolajczak et al., 2018 | N = 1723 | .31 | .31 | Medium |
|  | Endorsement by the coparent | Mikolajczak et al., 2018 | N = 1723 | -.26 | -.26 | Medium |
|  | Family disorganization | Mikolajczak et al., 2018 | N = 1723 | .45 | .45 | Large |
| Social support | Practical support | Lindström et al., 2011^a^ | N = 251 | .26 | .26 | Medium |
|  | Emotional support | Lindström et al., 2011^a^ | N = 251 | .22 | .22 | Medium |

*Note*. In order to make the resulting instrument widely applicable, we excluded from the Table risk/protection factors that would be only applicable to a minority of parents (e.g., functional level of the child for parents of a disabled child, Basaran, Karadavut, Uneri, Balbaloglu & Atasoy, 2013; perception of the severity of the disease for parents of a chronically ill child, Lindström et al., 2011). ^a^ In their paper, Lindström and colleagues used a dichotomous score of burnout. In order to get a more accurate estimation of the effect size, we contacted one of the authors of the study (Annika Lindahl Norberg) to get the correlation between the various antecedents investigated in their study and continuous scores of the burnout (SMBQ)’s total score. ^b^Aggregated correlations were computed using the online calculator “Averaging correlation coefficients” available on [www.stat-help.com](http://www.stat-help.com). **^c^** The interpretation of effect sizes was done according to Cohen’s “*d* equivalent to *r”* original norms for the social Science (Cohen, 1988). QS = Qualitative Study.

**Appendix B**

**Balance Between parental Risks and Resources (BR^2^) – English version**

Note for researchers: the first column indicates if the item is a common (C) or specific (S) antecedent. This column should be removed before giving the test to parents.

Please find below several questions about different aspects of your life. Click on the number that corresponds to your level of agreement with the statement from -5 to +5 (zero means that you cannot choose between statements)

|  |  | -5 | -4 | -3 | -2 | -1 | 0 | 1 | 2 | 3 | 4 | 5 |  |
| --- | --- | --- | --- | --- | --- | --- | --- | --- | --- | --- | --- | --- | --- |
| iiii  1C | I find it difficult to reconcile my family life and my professional life | -5 | -4 | -3 | -2 | -1 | 0 | 1 | 2 | 3 | 4 | 5 | I can easily reconcile my family life and my professional life. |
| 2222222  2 S | Because of my parental responsibilities, I can’t ever manage to find time for myself. | -5 | -4 | -3 | -2 | -1 | 0 | 1 | 2 | 3 | 4 | 5 | Despite my parental responsibilities, I easily manage to find time for myself. |
| é  3  C | I am quite stressed by nature | -5 | -4 | -3 | -2 | -1 | 0 | 1 | 2 | 3 | 4 | 5 | I am quite relaxed by nature. |
| -5  4  S | I feel that I don’t have the competences to be a good parent (e.g. I can’t get my children to obey me, I don’t know how to help them do well at school, I don’t know how to play with them) | -5 | -4 | -3 | -2 | -1 | 0 | 1 | 2 | 3 | 4 | 5 | I feel that I have the competences to be a good parent (e.g. I can get my children to obey me, I know how to help them do well at school, I know how to play with them). |
| -5  5  C | It is in my nature to aim at perfection (I put a lot of pressure on myself and/or I am afraid of how others see me). | -5 | -4 | -3 | -2 | -1 | 0 | 1 | 2 | 3 | 4 | 5 | It is in my nature to be tolerant of the mistakes I may make (I don’t put a lot of pressure on myself and I find others’ opinions worth listening to but not threatening). |
| -5  6  S | As a parent, I have high standards (I try to be a perfect parent, I put myself under a lot pressure and/or I am afraid of how others see me) | -5 | -4 | -3 | -2 | -1 | 0 | 1 | 2 | 3 | 4 | 5 | As a parent, I have normal standards (I am tolerant about mistakes I might make, I think other people's opinions are of interest but not threatening) |
| -5  7  S | I don’t share good times with my children (I don’t enjoy playing with them and/or they do not like the activities I suggest). | -5 | -4 | -3 | -2 | -1 | 0 | 1 | 2 | 3 | 4 | 5 | I share a lot of good times with my children (we like doing things together) |
| -5  8  S | I never take time to talk with my children (so they can tell me about their day, find solutions to their problems or exchange ideas and points of view) | -5 | -4 | -3 | -2 | -1 | 0 | 1 | 2 | 3 | 4 | 5 | I regularly take time to talk with my children (so they can tell me about their day, find solutions to their problems or exchange ideas and points of view). |
| -5  9  S | I don’t ask my children to be autonomous (I solve their problems for them to avoid conflict or because it’s easier etc.) | -5 | -4 | -3 | -2 | -1 | 0 | 1 | 2 | 3 | 4 | 5 | I encourage my children to be autonomous as soon as they can (to do their homework, dress themselves, travel back and forth, manage their pocket money, solve certain problems on their own etc. |
| -5  10  S | I never manage to say no to my children (I force myself to do things, I can’t refuse their demands, I am frightened of frustrating them) | -5 | -4 | -3 | -2 | -1 | 0 | 1 | 2 | 3 | 4 | 5 | I easily manage to say no to my children (I know how to convey my limits and needs, I know how to set them limits, I manage to refuse their demands). |
| -5  11  C | I can’t say no, I find it difficult to express my needs or say if something isn’t right for me. | -5 | -4 | -3 | -2 | -1 | 0 | 1 | 2 | 3 | 4 | 5 | I find it easy to say no, and to express my needs or say if something isn’t right for me |
| -5  12  C | When I express my emotions, I often hurt close family and/or friends | -5 | -4 | -3 | -2 | -1 | 0 | 1 | 2 | 3 | 4 | 5 | I can express my emotions without hurting close family and/or friends. |
| -513  13  C | I find it difficult to express my emotions. | -5 | -4 | -3 | -2 | -1 | 0 | 1 | 2 | 3 | 4 | 5 | I express my emotions easily. |
| -5  14  C | I naturally find it hard to manage my emotions (when I am angry, I explode; when I am sad, I have difficulties to cheer me up, …) | -5 | -4 | -3 | -2 | -1 | 0 | 1 | 2 | 3 | 4 | 5 | I normally find it easy to manage my emotions (when I am angry, I can express it calmly and constructively; when I am sad, I know what to do to cheer me up, …). |
| -5  15  C | It is in my nature to often feel negative emotions (sadness, anger, anxiety etc.). | -5 | -4 | -3 | -2 | -1 | 0 | 1 | 2 | 3 | 4 | 5 | It is in my nature to rarely feel negative emotions (sadness, anger, anxiety etc.). |
| -5  16  C | I think that what happens to me in life is due to chance or because of others' decisions. | -5 | -4 | -3 | -2 | -1 | 0 | 1 | 2 | 3 | 4 | 5 | I think that what happens to me in life depends on the choices and decisions I make. |
| -5  17  C | I’m afraid that my family and friends are not as fond of me as I am of them | -5 | -4 | -3 | -2 | -1 | 0 | 1 | 2 | 3 | 4 | 5 | I am convinced that my family and friends are as fond of me as I am of them. |
| -5  18  C | It is difficult for me to be intimate with my close relatives and friends (I can’t confide in them, I can’t hug them). | -5 | -4 | -3 | -2 | -1 | 0 | 1 | 2 | 3 | 4 | 5 | It is easy for me to be intimate with my close relatives and friends (I can confide in them easily and hug them to comfort them for example). |
| -5  19  S | I can never count on my relatives or friends to give me practical help with the children (babysit them, look after them, …). | -5 | -4 | -3 | -2 | -1 | 0 | 1 | 2 | 3 | 4 | 5 | I can easily count on my relatives or friends to give me practical help with the children (babysit them, look after them, …). |
| -5  20  S | When I have problems with my children, there is no friend or relative I can talk to about it (because I don’t have any or because I don’t dare or because they are not available) | -5 | -4 | -3 | -2 | -1 | 0 | 1 | 2 | 3 | 4 | 5 | When I have problems with my children, there are friends or relatives I can talk to about it (I dare to speak about it and they are available to listen to me). |
| -5  21  S | I have so many things to do for my children that I don’t have time to do everything. | -5 | -4 | -3 | -2 | -1 | 0 | 1 | 2 | 3 | 4 | 5 | I have time to do what is necessary for my children |
| -5  22  S | My children are so demanding of me that I don’t have a moment for myself | -5 | -4 | -3 | -2 | -1 | 0 | 1 | 2 | 3 | 4 | 5 | My children are demanding but I still have time free to do other things. |
| -5  23  S | I don’t have enough time to take care of my children | -5 | -4 | -3 | -2 | -1 | 0 | 1 | 2 | 3 | 4 | 5 | I have enough time to take care of my children. |
| -5  24  S | My partner and I do not agree at all about how to bring up the children. | -5 | -4 | -3 | -2 | -1 | 0 | 1 | 2 | 3 | 4 | 5 | My partner and I agree completely about how to bring up the children. |
| -5  25  S | My partner denigrates me as a mother/father. | -5 | -4 | -3 | -2 | -1 | 0 | 1 | 2 | 3 | 4 | 5 | My partner says that I am a good mother/father. |
| -5  26  S | My partner doesn’t help me at all with the children (he/she never looks after them, I feel as if I bring them up alone) | -5 | -4 | -3 | -2 | -1 | 0 | 1 | 2 | 3 | 4 | 5 | My partner helps me a lot with the children (he/she looks after them a lot, I can rely on him/her). |
| -5  27  S | My partner never listens to the worries I have as a parent. | -5 | -4 | -3 | -2 | -1 | 0 | 1 | 2 | 3 | 4 | 5 | My partner always listens to the worries I have as a parent. |
| -5  28  C | I feel very dissatisfied with my relationship with my partner. | -5 | -4 | -3 | -2 | -1 | 0 | 1 | 2 | 3 | 4 | 5 | I feel very satisfied with my relationship with my partner. |
| -5  29  C | My partner and I very often quarrel. | -5 | -4 | -3 | -2 | -1 | 0 | 1 | 2 | 3 | 4 | 5 | My partner and I never quarrel. |
| -5  30  S | At home, it is a real zoo (everything is disorganized, it's untidy, you can’t hear yourself think, we are always snowed under). | -5 | -4 | -3 | -2 | -1 | 0 | 1 | 2 | 3 | 4 | 5 | At home, we are well organized (the house is tidy, we find everything, we have routines etc.). |
| -5  31  S | I very often clash with my children | -5 | -4 | -3 | -2 | -1 | 0 | 1 | 2 | 3 | 4 | 5 | I never clash with my children. |
| -5  32  S | I am hyper-involved in my role as parent. | -5 | -4 | -3 | -2 | -1 | 0 | 1 | 2 | 3 | 4 | 5 | My role as parent is important to me but other things count more (my work, my leisure activities etc.) |
| -5  33  S | My partner is not at all involved in his/her role as parent. | -5 | -4 | -3 | -2 | -1 | 0 | 1 | 2 | 3 | 4 | 5 | My partner is hyperinvolved in his/her role of parent. |
| -5  34  S | I do not feel rewarded for my investment in my children (my partner and my children don’t realize what I do for them, they never say thank you). | -5 | -4 | -3 | -2 | -1 | 0 | 1 | 2 | 3 | 4 | 5 | I feel rewarded for my investment in my children (my partner and my children realize what I do for them, they very often say thank you). |
| -5  35  S | My view of bringing up children is completely different from that of other people who look after my child (teacher, baby sitter, grandparents, ex-husband/wife if applicable). | -5 | -4 | -3 | -2 | -1 | 0 | 1 | 2 | 3 | 4 | 5 | My view of bringing up children is similar to that of other people who look after my child (teacher, baby sitter, grandparents, ex-husband/wife if applicable). |
| -536  36  S | When I need time for myself, I can never count on my partner to look after the children | -5 | -4 | -3 | -2 | -1 | 0 | 1 | 2 | 3 | 4 | 5 | When I need time for myself, I can always count on my partner to look after the children. |
| -5  37  C | I am pessimistic by nature (the first thing I see is what’s going wrong, I automatically think that things will turn out badly). | -5 | -4 | -3 | -2 | -1 | 0 | 1 | 2 | 3 | 4 | 5 | I am optimistic by nature (the first thing I see is what is going well, I automatically think that everything will turn out well). |
| -5  38  S | My partner stresses me out as a parent (It’s more difficult to manage the children when he/she is there). | -5 | -4 | -3 | -2 | -1 | 0 | 1 | 2 | 3 | 4 | 5 | My partner makes me feel like a better parent (it is easier to manage the children when he/she is there). |
| -5  39  S | My children are always fighting (they bicker, they shout at each other, they gripe at each other…). | -5 | -4 | -3 | -2 | -1 | 0 | 1 | 2 | 3 | 4 | 5 | My children never quarrel (they never bicker, they never shout at each other, then never gripe at each other). |

**Appendix C**

**Balance Between parental Risks and Resources (BR^2^) – French version**

Note à l’attention des chercheurs: la première colonne indique si l’item est un antécédent commun (C) ou spécifique (S). Cette colonne doit être supprimée avant de donner le test aux parents.

Les énoncés ci-dessous sont présentés en opposition. Veuillez indiquer sur l'échelle de quelle phrase vous vous sentez le plus proche et à quel point. Le point du milieu signifiant qu'il est impossible pour vous de choisir la phrase vous correspondant le plus.

|  |  | -5 | -4 | -3 | -2 | -1 | 0 | 1 | 2 | 3 | 4 | 5 |  |
| --- | --- | --- | --- | --- | --- | --- | --- | --- | --- | --- | --- | --- | --- |
| iiii  1C | J’ai des difficultés à concilier ma vie familiale et ma vie professionnelle. | -5 | -4 | -3 | -2 | -1 | 0 | 1 | 2 | 3 | 4 | 5 | Je peux facilement concilier ma vie familiale et ma vie professionnelle. |
| 2222222  2 S | A cause de mes responsabilités de parents, je n’arrive pas du tout à trouver du temps pour moi. | -5 | -4 | -3 | -2 | -1 | 0 | 1 | 2 | 3 | 4 | 5 | En dépit de mes responsabilités de parents, je trouve facilement du temps pour moi. |
| é  3  C | De nature, je suis quelqu’un d’assez stressé. | -5 | -4 | -3 | -2 | -1 | 0 | 1 | 2 | 3 | 4 | 5 | De nature, je suis quelqu’un de plutôt détendu. |
| -5  4  S | J’ai le sentiment que je n’ai pas les compétences pour être un bon parent (ex : je n’arrive pas à me faire obéir de mes enfants, je ne sais pas comment les aider à réussir à l’école, je ne sais pas comment jouer avec eux). | -5 | -4 | -3 | -2 | -1 | 0 | 1 | 2 | 3 | 4 | 5 | J’ai le sentiment que j’ai les compétences pour être un bon parent (ex : j’arrive à me faire obéir de mes enfants, je sais comment les aider à réussir à l’école, je sais comment jouer avec eux). |
| -5  5  C | De nature, je suis quelqu’un de très perfectionniste (je me mets beaucoup de pression et/ou j’ai peur du regard des autres). | -5 | -4 | -3 | -2 | -1 | 0 | 1 | 2 | 3 | 4 | 5 | De nature, j’évite de me mettre trop la pression (je suis tolérant vis-à-vis des erreurs que je peux commettre, je trouve l’avis des autres intéressants mais pas menaçant). |
| -5  6  S | J’ai des standards élevés en tant que parent (je vise à être un parent parfait, je me mets beaucoup de pression et/ ou j’ai peur du regard des autres). | -5 | -4 | -3 | -2 | -1 | 0 | 1 | 2 | 3 | 4 | 5 | J’ai des standards normaux en tant que parent (je suis tolérant vis-à-vis des erreurs que je peux commettre, je trouve l’avis des autres intéressants mais pas menaçants). |
| -5  7  S | Je ne partage pas de moments positifs avec mes enfants (cela ne m’amuse pas de jouer avec eux et/ou les activités que je leur propose ne leur plaisent pas). | -5 | -4 | -3 | -2 | -1 | 0 | 1 | 2 | 3 | 4 | 5 | Je partage beaucoup de moments positifs avec mes enfants (nous prenons plaisir à faire des activités ensemble). |
| -5  8  S | Je ne prends jamais le temps de discuter avec mes enfants (pour qu’ils me racontent leur journée, chercher des solutions aux soucis qu’ils rencontrent, échanger des points de vue et idées ….). | -5 | -4 | -3 | -2 | -1 | 0 | 1 | 2 | 3 | 4 | 5 | Je prends régulièrement le temps de discuter avec mes enfants (pour qu’ils me racontent leur journée, chercher des solutions aux soucis qu’ils rencontrent, échanger des points de vue et idées ….). |
| -5  9  S | Je ne demande pas à mes enfants d’être autonomes (je résous les problèmes à leur place pour éviter les conflits ou par facilité etc… ). | -5 | -4 | -3 | -2 | -1 | 0 | 1 | 2 | 3 | 4 | 5 | J’encourage mes enfants à être autonomes dès qu’ils le peuvent (pour les devoirs, s’habiller, assurer leurs trajets, pour gérer leur argent de poche, résoudre seul certains problèmes, etc…). |
| -5  10  S | Je n’arrive jamais à dire non à mes enfants (je me force à faire des activités, je n’arrive pas à refuser leurs demandes, j’ai peur de les frustrer). | -5 | -4 | -3 | -2 | -1 | 0 | 1 | 2 | 3 | 4 | 5 | J’arrive facilement à dire non à mes enfants (je sais leur exprimer mes limites et mes propres besoins, je sais leur imposer des limites, j’arrive à leur refuser une demande). |
| -5  11  C | J’ai des difficultés à exprimer mes besoins ou à dire si quelque chose ne me convient pas. | -5 | -4 | -3 | -2 | -1 | 0 | 1 | 2 | 3 | 4 | 5 | Je n’éprouve aucune difficulté à exprimer mes besoins ou à dire si quelque chose ne me convient pas. |
| -5  12  C | Lorsque j’exprime mes émotions, je blesse souvent mes proches. | -5 | -4 | -3 | -2 | -1 | 0 | 1 | 2 | 3 | 4 | 5 | Je suis capable d’exprimer mes émotions sans blesser mes proches. |
| -513  13  C | Je suis quelqu’un qui a des difficultés à exprimer ses émotions. | -5 | -4 | -3 | -2 | -1 | 0 | 1 | 2 | 3 | 4 | 5 | Je suis quelqu’un qui exprime facilement ses émotions. |
| -5  14  C | De nature, je suis quelqu’un qui gère mal ses émotions (lorsque je suis en colère j’explose, lorsque je suis triste, j’ai des difficultés à me remonter le moral etc…). | -5 | -4 | -3 | -2 | -1 | 0 | 1 | 2 | 3 | 4 | 5 | De nature, je gère bien mes émotions (lorsque je suis en colère j’arrive à m’exprimer calmement, lorsque je suis triste je sais quoi faire pour me remonter le moral etc…). |
| -5  15  C | De nature, je suis quelqu’un qui ressent beaucoup d’émotions négatives. | -5 | -4 | -3 | -2 | -1 | 0 | 1 | 2 | 3 | 4 | 5 | De nature, je suis quelqu’un qui ressent peu d’émotions négatives. |
| -5  16  C | Dans la vie je pense que les choses qui m’arrivent sont dues au hasard ou aux décisions que d’autres prennent. | -5 | -4 | -3 | -2 | -1 | 0 | 1 | 2 | 3 | 4 | 5 | Dans la vie, je pense que les choses qui m’arrivent dépendent des choix que je pose et des décisions que je prends. |
| -5  17  C | J’ai peur que mes proches ne soient pas autant attachés à moi que je ne le suis à eux | -5 | -4 | -3 | -2 | -1 | 0 | 1 | 2 | 3 | 4 | 5 | Je suis convaincu que mes proches sont autant attachés à moi que je le suis à eux |
| -5  18  C | Il est difficile pour moi d’être intime avec mes proches (je n’arrive pas à me confier à eux, je n’arrive pas à les prendre dans mes bras) | -5 | -4 | -3 | -2 | -1 | 0 | 1 | 2 | 3 | 4 | 5 | Il est facile pour moi d’être intime avec mes proches (j’arrive facilement à me confier à eux, à les prendre dans mes bras pour par exemple les réconforter, …)  ). |
| -5  19  S | Je ne peux jamais compter sur mes proches ou mes amis pour m’aider concrètement avec les enfants (les garder, s’en occuper, …). | -5 | -4 | -3 | -2 | -1 | 0 | 1 | 2 | 3 | 4 | 5 | Je peux facilement compter sur mes proches ou mes amis pour m’aider concrètement avec les enfants (les garder, s’en occuper, …). |
| -5  20  S | Quand je rencontre des difficultés avec mes enfants, je n’ai aucun ami ou proche avec qui je peux en parler (parce que je n’en ai pas, ou je n’ose pas en parler avec eux, ou parce qu’ils ne sont pas disponibles). | -5 | -4 | -3 | -2 | -1 | 0 | 1 | 2 | 3 | 4 | 5 | Quand je rencontre des difficultés avec mes enfants, j’ai des amis ou des proches avec qui je peux en parler (j’ose en parler et ils sont disponibles pour m’écouter). |
| -5  21  S | J’ai tellement de choses à faire pour mes enfants que je n’ai pas le temps de tout faire. | -5 | -4 | -3 | -2 | -1 | 0 | 1 | 2 | 3 | 4 | 5 | J’ai vraiment le temps de faire ce qu’il faut pour mes enfants. |
| -5  22  S | Mes enfants me sollicitent tellement que je n’ai pas une minute à moi. | -5 | -4 | -3 | -2 | -1 | 0 | 1 | 2 | 3 | 4 | 5 | Mes enfants me sollicitent mais il me reste du temps pour faire autre chose. |
| -5  23  S | Je manque de temps pour m’occuper de mes enfants. | -5 | -4 | -3 | -2 | -1 | 0 | 1 | 2 | 3 | 4 | 5 | J’ai vraiment le temps de m’occuper de mes enfants. |
| -5  24  S | Mon/ma partenaire et moi ne sommes pas du tout d’accord sur la manière d’éduquer les enfants. | -5 | -4 | -3 | -2 | -1 | 0 | 1 | 2 | 3 | 4 | 5 | Mon/ma partenaire et moi sommes tout à fait d’accord sur la manière d’éduquer les enfants. |
| -5  25  S | Mon/ma partenaire me dénigre en tant que père/mère. | -5 | -4 | -3 | -2 | -1 | 0 | 1 | 2 | 3 | 4 | 5 | Mon/ma partenaire dit que je suis un bon père/ une bonne mère. |
| -5  26  S | Mon/ma partenaire ne m’aide pas du tout avec les enfants (il/elle ne s’occupe jamais d’eux, j’ai l’impression de les éduquer seul(e)). | -5 | -4 | -3 | -2 | -1 | 0 | 1 | 2 | 3 | 4 | 5 | Mon/ma partenaire m’aide beaucoup avec les enfants (il/elle s’occupe beaucoup d’eux, je peux me reposer sur lui/elle). |
| -5  27  S | Mon/ma partenaire n’écoute jamais les préoccupations que j’ai en tant que parent. | -5 | -4 | -3 | -2 | -1 | 0 | 1 | 2 | 3 | 4 | 5 | Mon/ma partenaire écoute toujours les préoccupations que j’ai en tant que parent. |
| -5  28  C | Je me sens très insatisfait(e) dans ma relation de couple. | -5 | -4 | -3 | -2 | -1 | 0 | 1 | 2 | 3 | 4 | 5 | Je me sens très satisfait(e) dans ma relation de couple. |
| -5  29  C | Nous nous disputons très souvent avec mon/ma partenaire. | -5 | -4 | -3 | -2 | -1 | 0 | 1 | 2 | 3 | 4 | 5 | Nous ne nous disputons jamais mon/ma partenaire et moi. |
| -5  30  S | C’est un vrai zoo à la maison (tout est désorganisé, c’est le bordel, on ne s’entend pas penser, on est tout le temps débordé). | -5 | -4 | -3 | -2 | -1 | 0 | 1 | 2 | 3 | 4 | 5 | A la maison, on est bien organisés (la maison est rangée, on s’y retrouve, nous sommes organisés, nous avons nos routines, etc…). |
| -5  31  S | Je suis très souvent en conflit avec mes enfants. | -5 | -4 | -3 | -2 | -1 | 0 | 1 | 2 | 3 | 4 | 5 | Je ne suis jamais en conflit avec mes enfants. |
| -5  32  S | Je suis hyper-investi dans mon rôle de parent. | -5 | -4 | -3 | -2 | -1 | 0 | 1 | 2 | 3 | 4 | 5 | Mon rôle de parent est important à mes yeux, mais d’autres choses comptent plus (mon travail, mes loisirs etc…). |
| -5  33  S | Mon/ma partenaire n’est pas du tout investi dans son rôle de parent. | -5 | -4 | -3 | -2 | -1 | 0 | 1 | 2 | 3 | 4 | 5 | Mon/ma partenaire est hyper-investi dans son rôle de parent. |
| -5  34  S | Je ne me sens pas récompensé pour mon investissement auprès de mes enfants (mon/ma partenaire et mes enfants ne se rendent pas compte de ce que je fais pour eux, ils ne disent jamais merci). | -5 | -4 | -3 | -2 | -1 | 0 | 1 | 2 | 3 | 4 | 5 | Je me sens récompensé pour mon investissement auprès de mes enfants (mon/ma partenaire et mes enfants se rendent compte de ce que je fais pour eux, ils disent très souvent merci). |
| -5  35  S | Ma vision de l’éducation est totalement différente de celle des autres personnes qui s’occupent de mon enfant (enseignant, gardienne, grands-parents, ex-conjoint si applicable). | -5 | -4 | -3 | -2 | -1 | 0 | 1 | 2 | 3 | 4 | 5 | Ma vision de l’éducation est similaire à celle des autres personnes qui s’occupent de mon enfant (enseignant, gardienne, grands-parents, ex-conjoint si applicable). |
| -536  36  S | Quand j’ai besoin de temps pour moi, je ne peux jamais compter sur mon/ma partenaire pour s’occuper des enfants. | -5 | -4 | -3 | -2 | -1 | 0 | 1 | 2 | 3 | 4 | 5 | Quand j’ai besoin de temps pour moi, je peux toujours compter sur mon/ma partenaire pour s’occuper des enfants. |
| -5  37  C | Je suis de nature pessimiste. | -5 | -4 | -3 | -2 | -1 | 0 | 1 | 2 | 3 | 4 | 5 | Je suis de nature optimiste. |
| -5  38  S | Mon/ma partenaire me stresse en tant que parent (c’est plus compliqué de gérer les enfants quand il/elle est là). | -5 | -4 | -3 | -2 | -1 | 0 | 1 | 2 | 3 | 4 | 5 | Mon/ma partenaire me réconforte en tant que parent (c’est plus facile de gérer les enfants quand il/elle est là). |
| -5  39  S | Mes enfants ne se disputent jamais (ils ne se chamaillent pas, ils ne se crient jamais dessus, ils ne se râlent jamais dessus, ...) | -5 | -4 | -3 | -2 | -1 | 0 | 1 | 2 | 3 | 4 | 5 | Mes enfants sont en dispute tous les jours (ils se chamaillent, ils se crient dessus, ils se râlent dessus, …) |
